# Lung ultrasonography for the diagnosis of 11 patients with acute respiratory distress syndrome due to bird flu H7N9 infection

**DOI:** 10.1186/s12985-015-0406-1

**Published:** 2015-10-26

**Authors:** Yu-kun Zhang, Jian Li, Jian-ping Yang, Ying Zhan, Jun Chen

**Affiliations:** Intensive Care Unit of the Department of Anesthesiology, The First Hospital Affiliated to Suzhou University, Suzhou, 215006 Jiangsu China

**Keywords:** Lung ultrasound, H7N9, acute respiratory distress syndrome (ARDS), multiple organ dysfunction syndrome (MODS)

## Abstract

**Background:**

A novel reassortant avian-origin influenza A (H7N9) virus was found to infect three Chinese residents, the first H7N9 infection in humans in Asia. Chest computed tomography (CT) for acute respiratory distress syndrome (ARDS) diagnosis is not only expensive but also exposes patients to radiation and might cause patients to be at risk of infection during transportation; in addition, chest radiography cannot be used to monitor the lung repeatedly in real time. Therefore, the routine use of bedside lung ultrasonography for critically ill patients with ARDS is especially valuable.

**Objectives:**

The aim of this study was to evaluate the application of ultrasound for lung examination in patients with ARDS.

**Methods:**

Eleven patients infected with H7N9 avian influenza who developed ARDS were diagnosed by lung ultrasonography.

**Results:**

Six patients who had severe ARDS showed a diffuse comet tail sign or a consolidation score ≥ 7 and a lung ultrasound score ≥ 20 points. A diffuse comet tail sign or a consolidation score ≤ 6 and a lung ultrasound score < 25 were observed in four patients. One patient showed a diffuse comet tail sign or consolidation area in four lung areas, with an ultrasound score of 14. Among all 11 patients studied, 6 patients had thoracic puncture and drainage of pleural effusion and 2 patients had pneumothorax puncture drainage.

**Conclusions:**

Lung ultrasound could be useful for monitoring ARDS caused by the influenza virus A H7N9 strain in clinical applications.

## Background

H7N9 is a novel avian influenza virus A strain whose infection of poultry occurs worldwide, but infection of this subtype in humans in Asia has not been observed previously [1]. Surprisingly, this strain was found to be highly pathogenic to humans and infected three urban residents in Shanghai or Anhui, China, in March 2013. The virus spread mainly though the respiratory pathway via direct or intimate contact with excretions or secretions from infected birds [2–6]. Since then, the new H7N9 strain infections in humans have spread rapidly from eastern China to other provinces of China, leading to approximately 130 cases of infections that are highly pathogenic, rapidly progress to the lower respiratory tract, and have a high mortality rate [2–6]. Infection by the H7N9 strain causes highly distributed lesions in the infected lung. Due to immune deficiency during the early-to-mid course of the disease, the alveoli of the infected lung excrete exudate accompanied by lung consolidation and/or pleural effusion [7]. Finally, most of the patients develop and some die of severe pneumonia, acute respiratory distress syndrome (ARDS), or multiple organ dysfunction syndrome (MODS) [8, 9]. Thus, it is extraordinarily important to evaluate accurately the pathological lung entities and pulmonary aeration in these patients.

Diagnosis and assessment of ARDS can be achieved by plain chest X-rays, ultrasonography, and computed tomography (CT). Chest CT is important for ARDS diagnosis but is expensive, exposes patients to radiation, and might cause patients to be at risk of infection during transportation [10–14]. Besides, chest radiography cannot be used to monitor the lung repeatedly in real time. Therefore, the routine use of bedside lung ultrasonography for critically ill patients with ARDS is especially important to monitor the chest changes in a timely manner for the diagnosis or treatment of the complications caused by infection of the H7N9 strain [4].

In this study, we reported the effect of treatment in patients infected with the H7N9 strain causing ARDS by observation of the lungs and pleura of patients using bedside lung ultrasonography.

## Materials and methods

### Study population

According to the 2012 Berlin ARDS diagnosis standards [15–18], 11 patients with ARDS were admitted to our Intensive Care Unit. Of the 11 patients (10 males, 1 female) with H7N9 pulmonary ARDS, one patient had mild ARDS, four patients had moderate ARDS, and six patients had severe ARDS. The average age of the patients was 59.09 ± 16.49 years old. Noninvasive mechanical ventilation (3 cases) and invasive mechanical ventilation (8 cases) were used. All patients were diagnosed and grouped according to the standards released by the National Health and Family Planning Commission of China. Laboratory confirmation of H7N9 virus infection was performed by reverse transcription polymerase chain reaction assays of throat swab samples or lower respiratory tract secretions.

### Equipment

The GE Healthcare Venue 40 ultrasound system (GE, USA) equipped with a convex array ultrasound probe (2–2.5 MHz) and a high-frequency linear array probe (5–13 MHz) was used in this study.

### Ultrasound examination

To avoid increasing hypoxemia by changing the body position, a semi-reclining position of the patients was kept during the ultrasound examination. Twelve lung regions were systematically examined:^2^ the upper and lower parts of the anterior, lateral, and posterior regions of the left and right chest walls by a sternal angle plane, anterior axillary line, and posterior axillary line. The anterolateral parts of the chest wall were examined in patients situated in the supine position, whereas posterior parts were examined in patients situated in the lateral position. Pleural effusion, alveolar consolidation, and alveolar–interstitial syndrome were observed. The 12 lung regions were explored and scored by three separate physicians, and the average of the 12 lung region scores was regarded as the lung ultrasonogram score for each patient. The scoring was performed according to the criteria described previously [15]. Normal ventilation (N, score of 1), A line; mild reduction of pulmonary ventilation, B1 (score of 2); severe reduction of pulmonary ventilation, B2 (score of 3); consolidation of lung signs (score of 5). The scoring was reproducible without major discrepancies, and ultrasonography (USG) assessment was repeated at least three times. Lung ultrasonograms were mainly patterned as a comet tail sign, consolidation, and pleural effusion. Artery blood gas analysis was performed, and the oxygenation index was calculated before the ultrasound examination.

### Data analysis

Experiments in each group were repeated three times. SPSS 13.0 software was used for the analysis. Data were expressed as means ± standard deviation ($\overline{x}$ ± *s*). Statistical significance among multiple groups was determined and analyzed by one-way analysis of variance, and comparison between the two groups was analyzed by the Student’s *t*-test using SPSS version 13.0 software. The *χ*^2^-test or the exact probability calculation method was used to compare the count data between groups. A *P*-value less than 0.05 was considered statistically significant.

## Results

### Patient clinical characteristics

All six patients with severe ARDS underwent mechanical ventilation (FiO_2_, 80–100 %; positive end expiratory pressure (PEEP), 15–20 cm H_2_O), and the mean oxygenation index was 65.6 ± 12.08. All these patients had a lung ultrasonogram score ≥ 20 and multiple comet-tail sign regions (or consolidation regions) with a score ≥ 7. There was no significant difference in the lung ultrasonogram score between patients with ARDS caused by H7N9 avian influenza infection and those with pneumonia caused by other viruses or bacteria [1].

### General patient data

Three patients quickly developed an unstable critical hemodynamic condition. Multiple comet-tail signs existed in 9–10 regions, including the area from the posterior back to the anterior chest. A restricted fluid infusion strategy, ventilation in the prone position, high frequency oscillator ventilation, and extracorporeal membrane oxygenation were used in the therapy, but oxygenation was not notably improved. The lung ultrasonogram scores in the lung areas in the present patients were ≥ 30 during the whole course of therapy. All three of these patients died at approximately two weeks after hospitalization. Multiple comet-tail sign region (or consolidation region) and lung ultrasonogram scores of the other three patients reduced markedly to ≤ 22 after the therapy.

### Prognosis results

**Fig. 1:**
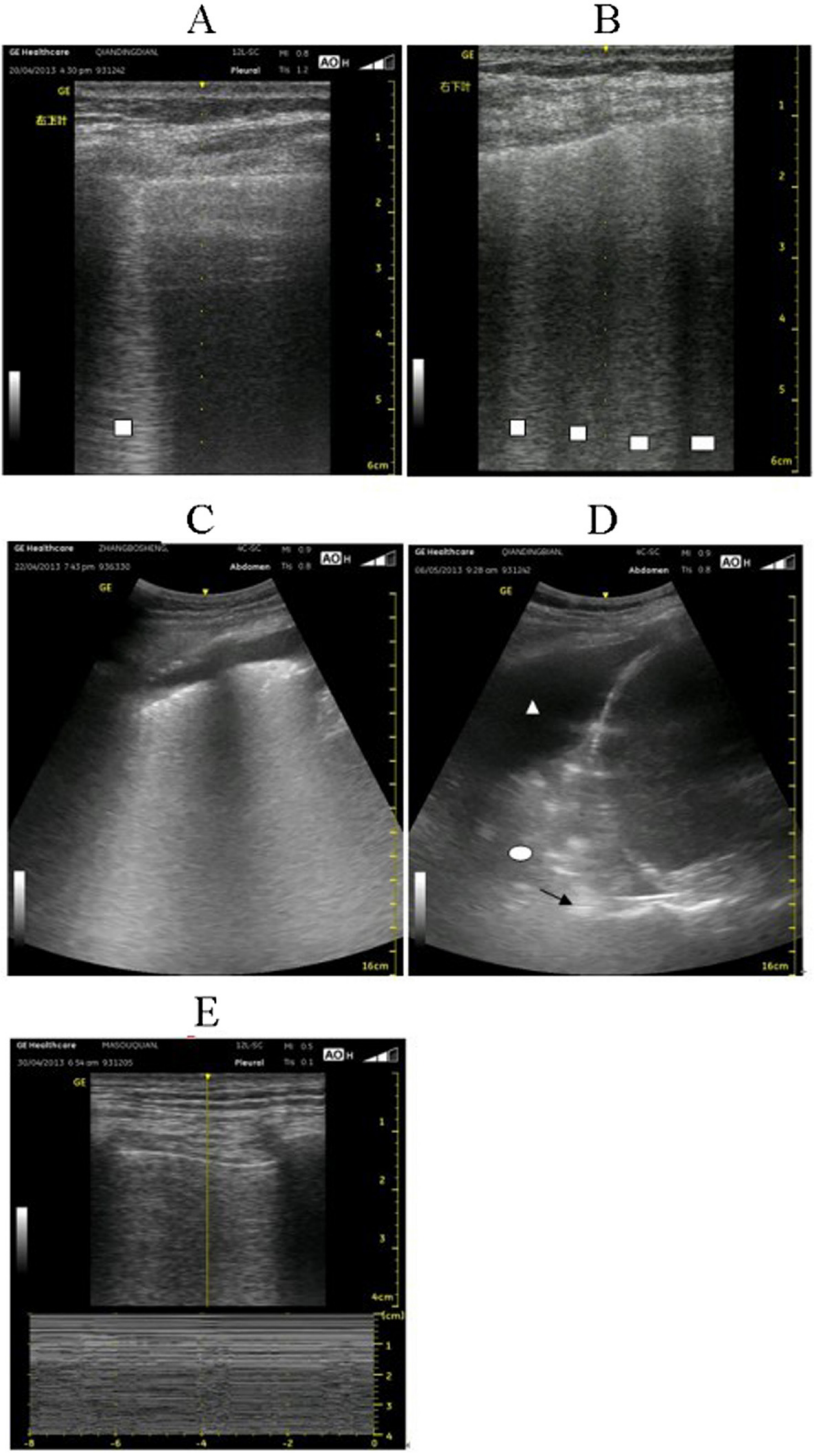
Lung sonography results. Patients with avian-origin influenza A (H7N9) virus were subjected to lung sonography analysis. □ B line; △ pleural effusion; ◯ consolidation; → air bronchography sign. **a** B line perpendicular to the pleura with a linear probe, namely, an isolated comet-tail sign. **b**: Four B lines with a linear probe, namely, a multiple comet-tail sign. **c**: Multiple comet-tail signs and pleural effusion with a convex array probe. **d**: Pleural effusion, lung consolidation, and air bronchography sign with a convex array probe. **e**: Disappearance of pleural sliding using a linear probe when the pneumothorax, stratosphere sign, and barcode sign were in the M modes

Two patients had recovered and were discharged from hospital. One patient had been successfully weaned from the ventilator and switched to nasal catheter oxygen for positive rehabilitation training. Four patients had pleural effusion and two had pleural effusion combined with pneumothorax, with one patient having a bilateral small amount of effusion, and the other case having a unilateral large amount of effusion (Fig. 1). The specimen obtained by thoracic puncture presented as mild bloody fluid. The oxygenation index after operation increased slightly, with a mean value of 82.66 ± 14.33.

Of the four patients with moderate ARDS, two underwent mechanical ventilation (FiO_2_, 60–80 %; PEEP, 8–10 cm H_2_O) and the other two underwent noninvasive mechanical ventilation (oxygen flow, 8–10 L/min; inspiration positive airway pressure, 15–18 cm H_2_O; expiration positive airway pressure, 6–8 cm H_2_O). The mean oxygenation index was 143.4 ± 29.76, the lung ultrasonogram score was < 25, and the number of multiple comet-tail sign regions (or consolidation regions) was ≤ 6 among these four patients. The ultrasonogram scores of these four patients were reduced to approximately 15 after therapy. One of the patients had a unilateral moderate amount of pleural effusion, and a thoracic puncture was performed accordingly. All four patients recovered and were discharged from hospital without requiring the use of a ventilator.

The patient with mild ARDS received a short period of noninvasive mechanical ventilation therapy. The oxygenation index was 260, the lung ultrasonogram score was 14, and the number of multiple comet-tail sign regions (or consolidation regions) was 4, without pleural effusion. This patient was discharged after therapy.

**Fig. 2:**
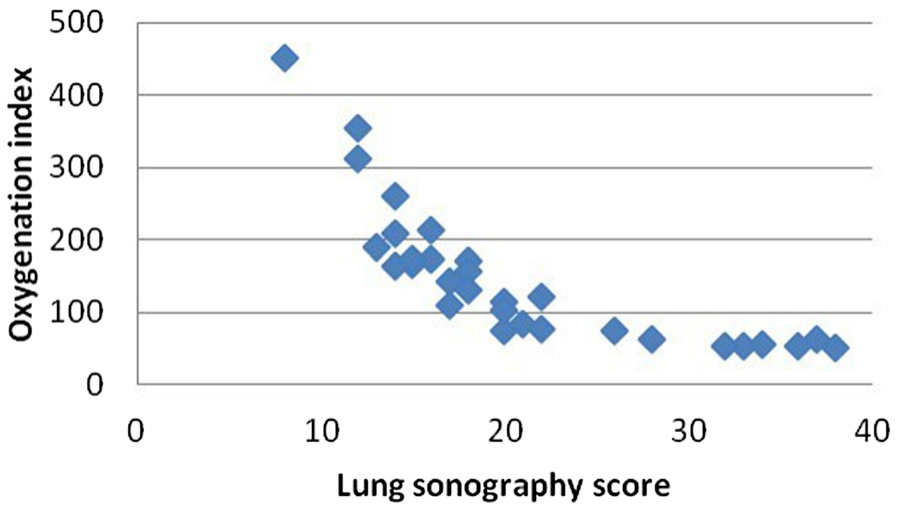
Relationship between the lung sonogram score and the oxygenation index. The correlation between the lung sonogram score and the oxygenation index was analyzed by Microsoft Excel (2007), and a negative correlation with a correlation coefficient of −0.782 is shown

The correlation between the lung sonogram score and the oxygenation index was analyzed by the CORREL function in Microsoft Excel (2007), and a negative correlation with a correlation coefficient of −0.782 was found (Fig. 2).

## Discussion

To determine the effect of treatment in patients with ARDS who had been infected with the H7N9 bird flu strain, we used bedside ultrasound to observe the lung and pleura of these patients. We found that bedside ultrasonography could be used to evaluate H7N9 avian influenza infection in patients with ARDS and could be useful in other clinical applications. Correlations between the lung ultrasound score, the oxygenation index, and the bedside ultrasound value in the influenza A (H7N9) ARDS patients were evaluated. The ultrasonography results showed that the treatment of patients with ARDS due to bird flu H7N9 infection was remarkably effective. There was no significant difference in the lung ultrasonogram score between patients with ARDS caused by H7N9 avian influenza infection and those with pneumonia caused by other viruses or bacteria. Based on our previous experience, we monitored the morphology of the lung and the intrathoracic airways as well as assessed the status of the lung ventilation and the interstitial thickening in the peripheral lung (in the absence of pneumothorax), and the results were consistent with previous investigations [15–18].

The lung ultrasonography of patients with ARDS showed not only a comet-tail sign but also exudation, consolidation, and atelectasis of the lungs as well as pleural effusion and pneumothorax [19–21]. The characteristics of ARDS in the patients infected with H7N9 were similar to previous investigations [19–21]; however, how to dynamically monitor the lung morphology and intrathoracic airways in patients in real-time and adjust the treatment plan for a definitive diagnosis has not been explored previously. We applied bedside lung ultrasonography to evaluate the lung injury by examining the pulmonary exudation and consolidation regions. We found that the lung ultrasonogram score was positively correlated with the severity of lung injury after H7N9 infection and that patients with severe ARDS had a greater score than other patients with multiple comet-tail signs, including the area from the posterior back to the anterior chest. Thus, the effective ventilation area reduced sharply, resulting in acute severe hypoxemia or even death. Therefore, we demonstrated that bedside lung ultrasonography can be used to observe the pulmonary morphological changes in patients infected with the H7N9 virus. In addition, the lung sonogram score and oxygenation index were negatively correlated, indicating that the sonogram score and oxygenation index similarly evaluated the patient’s condition during early ARDS. Although the statistical analyses had certain limitations due to the low number of cases and the short period of treatment, it was clearly shown that the mortality rate increased if the lung sonogram score continuously exceeded 30 in 1 week, which was closely related to exudation during early ARDS.

Pathological changes of the pleura can occur rapidly, even within hours after viral infection due to the critical virulence of the virus [19]. Pleural effusion usually emerges during the course of early-to-mid disease in accordance with the ARDS caused by H7N9 infection [22, 23]. The effusion volume might increase quickly in a short period. To maintain satisfactory oxygenation, a high PEEP value during ventilation was generally used, which possibly led to tension pneumothorax, especially when lung consolidation and pulmonary fibrosis developed. Once plural effusion or pneumothorax happened, the effective ventilation area decreased dramatically so that the hypoxemia was aggravated with a fatality [24, 25]. Therefore, bedside lung ultrasonography could help us to discover pulmonary morphological changes in a timely manner and guide the puncture accurately, leading to a favorable prognosis.

Although not proven, the possibility of human-to-human transmission of the H7N9 virus cannot be ruled out. Transporting patients infected with H7N9 out of hospital may lead to the spread of infection. Thus, bedside examination, including ultrasonography, can contribute to reducing the chance of nosocomial infection and transmission.

The ultrasonography results showed that the treatment of patients with ARDS due to bird flu H7N9 infection was remarkably effective. The treatment options and this conclusion were based on our previous experience.

## Conclusion

In conclusion, bedside ultrasound can be used to evaluate H7N9 avian influenza infection in patients with ARDS with pulmonary and pleural lesions, providing a strong basis for adjusting the treatment plan for patients. Therefore, lung ultrasound could be useful for the timely monitoring of ARDS caused by the H7N9 virus in clinics where H7N9 infection is emergent in humans.
